# “Figuring out how to be normal”: Exploring how young people and parents make sense of voice‐hearing in the family context

**DOI:** 10.1111/papt.12381

**Published:** 2022-01-20

**Authors:** Claire Mayer, Guy Dodgson, Angela Woods, Ben Alderson‐Day

**Affiliations:** ^1^ Department of Psychology Newcastle University Newcastle upon Tyne UK; ^2^ Cumbria, Northumberland, Tyne and Wear NHS Foundation Trust Newcastle upon Tyne UK; ^3^ Institute for Medical Humanities, Pharmacy and Health Durham University Durham UK; ^4^ Department of Psychology Durham University Durham UK

**Keywords:** adolescence, family, interpretative phenomenological analysis, sense‐making, voice‐hearing

## Abstract

**Objectives:**

Making sense of voice‐hearing—exploring the purpose, cause, and relationship with voices—is seen as therapeutically valuable for adults, but there is a paucity of research with adolescents. Family intervention is recommended for young people, yet little is known about families’ perspectives on, or role in, a child's voice‐hearing. This study therefore aimed to explore how both young people and parents had made sense of voice‐hearing in the family context.

**Method:**

Semi‐structured interviews were conducted with seven young people who hear voices (six females, one male, age *M* = 17 years) and six parents of young people who hear voices (five females, one male). Data were analysed using interpretative phenomenological analysis.

**Results:**

The young people struggled to reconcile their voice‐hearing experiences within themselves, wanted control, ‘normality’, and not to let their mental health hold them back. Parents saw the voices as separate to their child, who they were protective of, and came to an acceptance and hope for the future amidst continued uncertainty. Pragmatism, and shame, ran through parents’ and young people's accounts. Tensions between them, such as autonomy versus involvement, were also apparent.

**Conclusions:**

Few participants had made sense of their experiences in any concrete form, yet hope, control, and getting on with their lives were not conditional on having done so. Young people valued the family as a safe, non‐enquiring space to be ‘normal’ and *not* to talk about their experiences. While all had been challenged by their experiences, an energy and strength ran through their accounts.

**Practitioner points**

- Feeling ‘normal’, in control and getting on with life were of primary importance to the young people, and not conditional on having made sense of voice‐hearing, which few had done.
- Family was valued by young people as a place to be ‘normal’ and *not* to talk about voices.
- Connecting with others with similar experiences was important for young people and parents, but opportunities to do this had been lacking.
- Parents would benefit from their own therapeutic space to process their experiences and to develop their understanding of their child's difficulties and how best to support them.

## INTRODUCTION

A bio‐medical paradigm tends to view voice‐hearing, or ‘auditory verbal hallucinations’, as a symptom of a serious mental ‘disorder’ such as schizophrenia (American Psychiatric Association, 2013). An alternative view sees voice‐hearing as being on a continuum of normal human experience (Corstens et al., 2014), regularly experienced by between 5% and 15% of adults (Beavan et al., 2011), and around 12% of children and adolescents (Maijer et al., 2018). From this perspective, voices can be construed as holding significance and value for the individual rather than being simply a symptom to manage (Woods, 2013). Multiple pathways to voice‐hearing have been identified (Luhrmann et al., 2019) and ‘listening to the voices’ (Longden, 2017, p. 573) recommended, notably by those in the Hearing Voices Movement (Corstens et al., 2014).

Making sense of one's experiences, reflecting on what has happened, and understanding and interpreting its significance and purpose, is an essential human endeavour (Smith, 2019). In the context of voice‐hearing, it is seen to be therapeutically valuable (British Psychological Society, 2017) by improving quality of life (Lonergan, 2017) and decreasing shame and stigma (Romme et al., 2009). A cognitive model of voice‐hearing foregrounds appraisals of voices such as purpose and cause (Chadwick & Birchwood, 1994), linking these to distress and coping strategies (Sayer et al., 2000). The hearer's relationship with their voices, particularly power and agency, is also highlighted. Holt and Tickle’s (2014) synthesis of studies of adult voice‐hearers’ first‐hand accounts highlights commonalities in their personifying of voices, including the importance of actively engaging with voices and of challenging their perceived power. Other studies highlight the range of individuals’ causal explanations for their voices, including spiritual and religious interpretations, and those relating to life experiences and ‘mental illness’ (e.g. Jones et al., 2003).

How far sense‐making relates to young people's voice‐hearing is unclear. Voice‐hearing in adolescence can be particularly distressing (Bartels‐Velthuis et al., 2011), has links to suicidality (Jardri et al., 2019), and is a risk factor for psychosis (Welsh, 2013). In Kapur et al.’s (2014) survey with young voice‐hearers using mental health services (age *M* = 17 years), 21 had some certainty as to why they heard voices and nine did not know, though unfortunately their beliefs were not explored further. Escher et al. (2004, 2002) interviewed 80 young people (age range = 8–19 years), half of whom were receiving support; 75% linked their voice‐hearing to an experience of powerlessness. Parry and Varese (2020) surveyed 68 young voice‐hearers (age *M* = 14.91 years), 36% of whom had accessed services. Many described positive aspects of voices such as companionship and comfort. Most experienced a loss of control over their emotions and actions overall. Attributing voices to past experiences or personal qualities was seen to reduce distress and voices’ power. This is a mixed picture, particularly in a clinical context, and the lack of research into young people's voice‐hearing is consistently highlighted (Maijer et al., 2019).

Sense‐making takes place in a social context. In services for young people, the context of the family is privileged; family intervention is recommended by the National Institute for Health and Care Excellence (2016) and in early intervention in psychosis (EIP) guidelines (NHS England, 2016) based on evidence from adults (National Collaborating Centre for Mental Health, 2016). However, our knowledge of families’ experiences is limited. Family members may be struggling to understand a loved one's voice‐hearing (Hayward & Fuller, 2010) and grappling with their own emotional response (Escher & Romme, 2010). Parry and Varese’s (2021) survey with 132 parents described experiences of strain and a lack of support. Kapur et al. (2014) surveyed 27 parents and interviewed 2 in depth; 78% had some certainty of why their child heard voices and 22% did not know. Half characterized how they felt as ‘lost’.

To inform therapeutic work with young people who hear voices, including the shape of potential family involvement, greater understanding is needed of how young people and their families have been able to make sense of voice‐hearing, both individually and together. This study therefore took a broad aim, to explore how young people and parents had made sense of voice‐hearing in the family context.

## METHOD

### Design

Interpretative phenomenological analysis (IPA; Smith et al., 2009) was selected as the methodology due to its emphasis on working up from rich exploration of individual cases, its applicability to sense‐making (Smith, 2019) and its ability to explore both convergence and divergence in perspectives.

A two‐group qualitative design was used. The first group were young people aged 12–19 who hear or have heard voices for more than a month and had received support for this from mental health services. The second group were parents of young people who had started hearing voices aged 12–19. The groups were separate, with young people and parents not required to be related.

### Procedure

Participants had to be able to talk about their experiences in an interview of up to 60 min, have capacity to consent or assent, and be fluent in English. They were selected through purposive sampling (Creswell, 2013) through contact with staff in EIP teams and children and young people's services in two mental health trusts in Northern England.

Data were collected by the first author in face‐to‐face semi‐structured interviews. Participants choose where to be interviewed. Interviews lasted between 45 and 90 min and were conducted between July and December 2019. Participants were given a £20 voucher for their time. Written informed consent was obtained before each interview. Afterwards, participants were given a debrief sheet detailing sources of information and support. Interviews were audio‐recorded and then transcribed verbatim by a transcriber. Names and identifying information were removed at this stage to protect confidentiality.

### Materials

The method was developed in consultation with a parent and young person with experience of voice‐hearing. Separate interview schedules for young people and parents covered voice‐hearing experiences, ideas on origins of these, how voice‐hearing had been discussed in the family, impact on family relationships and participants’ advice to other young people, families, and health professionals. A family sculpt exercise (Dallos & Draper, 2010) was used as required to aid discussion. Further details are included in Supplementary Materials.

### Participants

Eighteen potential participants were approached; thirteen agreed to participate. Details are shown in Table 1. All participants lived with their parent or child. All parents were in their 40s and 50s. One parent and one young person were from a British Bangladeshi background, and the remainder were White British. There were three pairs of related parents and young people in the sample; to preserve anonymity they are not presented or analysed as pairs.

**TABLE 1:** Details of participants

Young people			
Pseudonym	Age	Where interviewed	EIP or CAMHS^a^
Zoya	16	NHS	CAMHS
Adelle	17	NHS	CAMHS
Tom	19	NHS	EIP
Hannah	17	School	CAMHS
Lucy	17	NHS	CAMHS
Sarah	18	Home	EIP
Jade	18	Home	EIP

Parents			
Pseudonym	Age and gender of child^b^	Where interviewed	EIP or CAMHS
Anna	Age range 16–24 years Age *M* = 18 years Female = 3 Male = 3	Home	CAMHS
Emma		Home	EIP
Jackie		Home	EIP
Peter		University	EIP
Fatima		University	CAMHS
Maria		Home	EIP

^a^ Child and Adolescent Mental Health Services.

^b^ Details not matched to participants to preserve anonymity between related parents and young people.

### Analysis

IPA analysis (Smith et al., 2009) was conducted by the first author. It took place firstly for each participant, noting initial thoughts on the audio recording and transcript. Detailed examination of conceptual, descriptive, and linguistic features and the first author's emerging interpretations followed, from which themes and summary narratives for each participant were developed. Convergence and divergence were explored within each group and subordinate and superordinate group themes developed with reference to the individual accounts. This was iterative, going between transcripts, individual and group analysis, and writing, with final themes agreed by the team. Analysis was aided by NVivo12 for Mac. The sample size is consistent with similar studies (e.g. Milligan et al., 2013).

The research was approved by an NHS ethics board and the two participating NHS trusts.

## FINDINGS

Findings are presented in the groups of young people and parents. Most themes were reflected in all accounts in the group; where themes were not universal, they were only absent from one participant's account.

### Young people findings

Analysis of the interviews with young people produced four superordinate themes: ‘The voices and me’, ‘Working out what's going on’, ‘I’m still me’, and ‘Being me and in my family’. These reflect the struggle to understand the voices as a part of the self, the influences of health professionals and family stories, accommodating the voices to get on with being ‘normal’, and the dynamics within families.

#### The voices and me

##### Is this me?

The young people talked powerfully of their voices as both part of them and an ‘other’ that could be ‘scary’ (Lucy) and ‘intense’ (Zoya). Each had reflected on their voices, sometimes seeing elements of themselves in gender, tone, and content, sometimes not. The strongest sense of an ‘other’ was when voices told them to hurt those they loved:

Sometimes I’d feel like it is me, because obviously I was depressed and did want to die, and I was like maybe it is me, because I want to die and it's telling me to die and I hated myself, I just hated myself…it was so confusing…but then when it came to harming my family, I was like I would never do that. (Zoya)

Some rejected personifying their voices and the idea of a more formed ‘other’ as a way of retaining control; ‘I don't want to give them names but that's…I don't know, it's just kind of like…because then it's more like they're people, and I know they're not people, which would just confuse my brain even more…They're just voices’ (Adelle). Others were more curious and exploratory; ‘I heard like a voice and I was like…that man looks like…sounds like he'd be bald! It sounds like he'd be bald and he'd be wearing a polo top. That's sometimes what I…imagine it to be’ (Lucy).

The relationship with voices was complex and shifting. Hannah's could be positive and helpful; Zoya and Lucy's helped them feel less lonely, while Sarah's was ‘helping me, keeping me safe’.

##### A struggle for control

All had felt controlled by their voices which had told them to self‐harm or to hurt other people, and felt that the voices had interfered in relationships and activities: ‘He tells us to push everyone away and it's…I have like no mates. Ehm, he tells us what to do and I listen to him sometimes’. (Jade); ‘It's like being back in school and you're getting bullied, but you cannot punch the bully in the face…you can't lay your hands on them ‘cos it's not real’ (Tom).

Half described ways of regaining control.

They do still like…like appear sort of thing and I just acknowledge them and I’m like, OK, well do I find value in what they are saying sort of thing? So I feel as though at the moment I’m coping quite well. (Hannah)

This remained, however, in the context of an ongoing struggle.

#### Working out what's going on

##### I was told that…

Explanations offered by health professionals dominated the accounts. These usually remained in the language of the professionals, hinting at their limited internalization. Tom valued his psychologist's explanations of inner speech. Zoya had been told they were ‘intrusive thoughts… But then I don't know what it really means!’ For Lucy, ‘They've just kind of explained that it happens and that it's OK. But they haven't really explained why it happens, why it's there’.

Hannah had been told her voices were linked to a bereavement. It had been suggested to Tom and Jade that witnessing domestic violence could be a factor. Adelle spoke of her ‘backpack full’ of ‘trauma’. However, none were fully persuaded by these explanations and struggled to connect them with their experience of voices: ‘If the traumas were the cause, then how did it take so long for it to kind of … take off?’ (Adelle).

##### A label that fits?

Voice‐hearing was intertwined with other mental health issues, and there was no neat diagnostic ‘fit’. Tom was the only one to speak of having a ‘mental illness’. For Jade, a diagnosis of psychosis was, for now, sufficient explanation, in the absence of anything else. Tom felt psychosis could be ‘cured’ so was comfortable with this, while wanting to avoid a label of schizophrenia, for which he saw no ‘cure’. Hannah and Zoya welcomed confirmation they did not have psychosis or schizophrenia as that would be ‘serious’. All identified with depression and anxiety, which felt tangible and socially acceptable; ‘Like someone says they've got depression, it's like oh everyone feels sorry for them but if someone's got voice‐hearing…you're a nutter’ (Tom). All were, first and foremost, looking for confirmation that they were not ‘crazy’ (Sarah), ‘nuts’ (Jade), or ‘insane’ (Lucy).

##### Family stories

Sarah and Jade mentioned grandparents with schizophrenia. Even though they had passed away, tales had travelled down the generations; ‘Me mam did say like he was mental! Like he was writing on the walls and all that’ (Jade).

Many spoke of their mums’ mental health, of wanting to protect and not ‘worry’ them, or of being self‐sufficient as they felt their mum would not, or could not, support them.

Families had set the tone for how mental health was perceived. Some viewed it with fear, one with empathy and care, but for most it simmered unspoken under the surface. Zoya had struggled with her family's beliefs about mental health, and appreciated their attempts to look at different perspectives:

It was just like you know them [religious leaders] coming and saying prayers, it just made you feel like so … that you’re the problem in the house, do you know what I mean? Like a big problem towards them. And I felt very bad about that. Because I thought I was possessed like, like everyone …at that time I didn’t know.

Zoya's was the only interview where the family's influence was a strong focus; for others, family was talked of as incidental, their influence absorbed almost unconsciously.

#### I’m still me

##### Accommodating this as me

Participants drew on experiences of being bullied, childhood difficulties, bereavements, diagnoses, and a sense of having a special gift to try to make sense of their experiences. Only Sarah's narrative felt fully formed in finding ‘a sense of myself’. Lucy, who had heard voices since childhood, was resigned:

Like I used to want to get rid of it, and I didn’t want it to be there, but now I’m just kind of like…I’m fine that they’re being there as long as it’s not horrible…It’s like part of me.

Finding other meaning had helped some. Most felt closer to their family. Sarah's new job was connected to her voice‐hearing. Zoya felt ‘What I went through made me much stronger’.

Clarity was not a prerequisite for getting on with life and most accommodated the uncertainty and ambiguity: ‘I mean I don't know…I would love to know why! I’d love to know like what I’ve done or like…why it's happening, but now I just focus on what to do’. (Tom). ‘Me’ continued no matter what.

##### Being normal

A need to feel “normal” (Tom)—to do and want what teenagers do and want—grounded the young people and gave a sense of forward motion. All but one felt they were moving towards a future they wanted and most continued to go to college or work, saw friends, and wanted to live independently:

I’ve tried to get on with life because you've got to get on with life in the end, like life's not going to stop for you, like you've got to try and find a way of making life work for you. (Sarah)

Seeing voices as ‘normal’ was a comfort. Just knowing others had similar experiences was helpful, while some had met others in person or on‐line in what Sarah called her ‘safe space’.

The opportunity to talk to health professionals alone or with family had been a containing, validating, and boundaried space to ‘let it out’ (Jade) in ‘the only place I’ve ever talked about it’ (Lucy). ‘I see [Care Coordinator] once every week. It's like they're the times for us to talk about it and that and the rest of the time it's just figuring out how to be normal’ (Tom).

#### Being me and part of a family

##### Doing it myself

All the young people wanted to get on with life independently of their parents:

I think it might be part way an age thing because like it's come to the point where I’m just like I want to do a lot of things on me own… …It's like knowing that I have gotten through things before meself, so I could get through it again. (Hannah)

None had told family about their voices first; five had told health professionals and the other participant told friends. They carefully calibrated who to talk to based on a blend of shame, fear of others’ reactions and attitudes, anticipated and actual lack of understanding and support, and wanting to protect others.

While families’ efforts to understand were appreciated, a profound sense of loneliness prevailed. ‘He [dad] doesn't get it, like he doesn't get anything about mental health’ (Lucy). Only Sarah had found a way she was comfortable with to understand her experiences and to explain them to her family in ways that made sense to them; ‘They [my grandparents] know that…I’ve got a poorly mind and stuff and I have anxiety and I have a bit of depression and stuff’ and ‘She [little sister] knows I went to hospital because I was ill but my brain was ill, it wasn't me that was ill, it was just my brain’.

##### Family as normality

While existing good relationships with parents had been enhanced, difficult ones tended to be exacerbated, even if they offered practical help:

Obviously she's supportive and like she comes to appointments and stuff, but now I just, I just come by meself, it's just easier because she just gets upset, because she doesn't really understand. (Adelle)

All the young people had found comfort with at least one parent, not of understanding but of a space that was ‘normal’ and accepting, enabling them to ‘just be’. Adelle watched TV with her dad, while Lucy and Sarah's dads made them laugh. Grandparents and siblings featured heavily in this making of ‘normality’, even if they did not ‘get it’; older siblings away from home provided a space to retreat to while the dynamics with others continued:

I’ve never spoke to him [my little brother] about it. I try and … to not let him see me when I’m at me worst. ‘Cos I don't want him to think his brother's a loonie! (Tom)

### Parent findings

Analysis of parents’ interviews produced three superordinate themes: ‘Trying to understand’ explores parents’ relationship with the voices their child, and their search for an explanation; ‘Protecting my child’ details how, in the context of uncertainty, parents were proactive and protective; and ‘Holding yesterday, today and tomorrow’ describes their continued hope for their child's future, and the personal meaning of their experiences.

#### Trying to understand

##### Not my child

All parents saw the voices as separate from their child, referring repeatedly to an ‘other’, whether a ‘powerful brain’ (Peter), ‘the head’ (Fatima), the ‘intrusive thoughts’ (Anna) or ‘the voices…it just wasn't my daughter, that's the only best way I can describe it’ (Emma).

This ‘other’ was a target for anger and blame for their child's pain and distress, distancing their child from the intensity of experiences described as ‘dangerous’ (Maria), ‘frightening’ (Emma) and ‘out of control’ (Anna). None had personified the voices beyond their child's descriptions, with Anna actively rejecting this: voices belong to people, these did not, so they were just to be treated as ‘intrusive thoughts’. They were not her child.

##### Searching for the why

Parents’ explanations were often only emerging and voice‐hearing meshed with other aspects of their child's experiences. Biological explanations were prevalent; for Peter it was ‘the brain’, for Fatima a ‘disease’, and for Jackie and Maria an ‘illness’. Only one talked of a diagnosis (of psychosis) having had explanatory value. All talked of ‘anxiety’ while others mentioned ‘stress’ (Fatima) and a ‘breakdown’ (Emma).

Participants connected the voices with bullying, exam pressure, bereavement, physical illness, sexual harassment, drug use, domestic violence, and the spiritual. Most had questioned if they had done ‘something wrong’ (Emma). Only a few were solid in their—very different—conclusions:

Looking at, you know, kind of the brilliance of having an imaginative mind that could create all of this as well, I look at that quite positively, not negatively, it's not … it's not a bad thing, you know it has kind of … you know its darker areas that can be a challenge sometimes, but actually you know overall … that … that kind of creative mind is really quite wonderful and quite special. (Anna)

He's got an illness like any other illness, and as far as I’m concerned, that's nothing to be ashamed of. It's happened, it's happened for a reason, we don't know why … unlucky. You can get hit by a bus, you don't know why you've been hit by a bus. (Jackie)

For most, the search still continued.

#### Protecting my child

##### Keeping you close

All but one parent described wanting to keep their child physically close, expressing love while mired in fear and uncertainty, and when talking could be difficult: ‘She [my daughter] used to cry and say, “I’m sorry, I’m putting people through this thing, send me away”. I said, “no I will not send you away, we're going to fight together as a family”’ (Fatima). Jackie described a brutal break in this proximity when her son was sectioned, highlighting that proximity was not always in parents’ hands. A number recalled times of being on constant watch for their child, while others talked of their struggle to let go as they recovered.

While parents’ instincts were to move towards their children, they were conscious that their child's instinct was often to move away, taking refuge in their bedroom, being with friends, meeting health professionals on their own, and not wanting to talk about what was happening. Parents held this tension as one of a teenager's natural desire for space, but with difficulty: ‘Part of the problem is now they're eighteen and they don't want them hugs and stuff like that’ (Maria). Finding a balance between closeness and separateness was fluid.

##### I've got your back

The mothers were strongly protective: ‘She [my daughter] knows I’m always here’ (Emma). They talked, listened, helped with medication, tried to give space, supported future plans, soothed, and laughed:

Giving her a hug and spending time talking, taking out to town for coffee, spend more time with her, talking to her. Sometimes she'll put her head in my lap, so I’ll massage her head. When the voice talking, so I’ll speak to her, massage her…massage her face. (Fatima)

Peter's care, meanwhile, was shown through a strong desire to help his son, now in his mid‐20s, get on with his life and get through the difficulties he faced.

Half the participants had confronted their child's voices directly. Jackie had argued with her son's voices, while Peter described being in ‘competition’ with them. In the context of an overwhelming feeling of powerlessness, Maria's strongest moment was when she confronted her daughter's voice:

I went ‘put him [the voice] on the phone!’ Ehm, and it’s not like if somebody … I don’t know how to describe it, it’s not like somebody’s answering us back, she doesn’t have this voice playing back if I’m talking. But I sat on the phone and I went, ‘you’re nobody, you know, my daughter’s beautiful’. I starting giving that, I said, ‘look at you, you’re ugly, she’s better, she’s, you know, she’s no failure’ and I start like arguing with this voice.

Anna and her son had successfully ‘belittled and ridiculed’ his voices together and she described strategies they had found to take back ‘power and control’. Each parent had found their way to continue to protect.

##### Drawing a protective circle

Parents were conscious of their child being judged by others; ‘I think people just…look at that [psychosis] and think maybe you know… different views of schizophrenia and things like that, you know what I mean, is she dangerous, is she this, she's that?’ (Maria). One mother had to confront her community's negative ideas about mental health, while Peter recalled taking his distressed son for a drive so the neighbours would not see.

All had drawn a small, protective circle around their family, restricted to those who would ‘understand’. Emma had not told her friends and did not want her mum to see ‘all the bad bits’ while Peter reflected ‘I’m not keeping it secret, but I’m not burdening other people either’.

I wouldn't allow anybody to judge him. Anybody negative want to come in and start calling him names, they'll just have to go, they just wouldn't be a part of wah [our] lives, because he's more important’. (Jackie)

The underlying sense was of a parent's duty to ‘just to get on with it’. Participants rarely spoke explicitly of isolation. Nevertheless, isolation reverberated through their words; ‘not that it was kept under wraps, but like I just thought, this can be sorted in this house, this can be…I can like deal with everything’ (Emma). Within immediate family there were layers of understanding and things unsaid. Half talked of supportive conversations with their partner, but, for two, their partners felt emotionally absent and only Fatima mentioned the involvement of siblings. Trying to protect their child from society's potential judgement was a heavy burden.

#### Holding yesterday, today, and tomorrow

##### Uncertainty and acceptance

All had reached an acceptance of their child's voices as somehow part of, yet separate to them: ‘I don't know what I think [the voice is]. To be honest, I think it's [son's name]. There's no other way I can explain it, it's [son's name]’ (Jackie). ‘Whatever you hear, it's not real, it's just imagination and you see, it's…nothing is real, you just don't have enough chemical in your brain’ (Fatima). ‘Now she does understand, and even I do, because you know the voices that she kept hearing…it was nobody, it was her voice she could hear in her head’ (Emma).

This was despite a lingering lack of certainty. The parents were disappointed that answers from health professionals felt promised but never came. Peter and Fatima held on to hope that their children would ‘grow out’ of the voices, while Jackie and Anna accepted a likely permanence. Emma's daughter's voices were now infrequent but she worried ‘it’ would return and still kept a window that her daughter had climbed out of when she was unwell locked, just in case.

For some, acceptance related to a greater sense of control: ‘There are strategies, there are things that work [against the voices], that are things that help and I think particularly taking some power and control over it’ (Anna). ‘I do everything I can’ (Jackie). Empathy was also key; Peter talked of his son's ‘whipping’ and ‘flagellation’ of himself, while for Maria, ‘Just to hear that [the voices] all the time constantly I think must be hard, really hard’. It helped the uncertainty to be held.

##### Pragmatism and hope

Forward momentum came from taking ‘one day at a time’ (Peter), overcoming ‘hurdles’ (Emma), and taking ‘baby steps’ (Jackie). These could be as significant as passing exams, or something as everyday as a drive, a game on the X‐Box, or learning to cook pasta in preparation for university.

Some were philosophical; ‘When somebody's really poorly, this is the peak, this is the top of the mountain. And it doesn't go on forever and it will get better’. (Anna). ‘It's like I know…she knows it's happened and like she says, “mam, me life's like now, present, future, not what's gone on”’ (Emma). All held hope for their child and their future:

All I care about is it doesn't define him. I don't want this to be his life. I want him out there, I want him socialising, I want grandkids, I want girlfriends braying at me door, telling us he's been a git! I want all of that for [name], as any [age] year old should be living’. (Jackie)

Maria's daughter feels more ‘stuck’ but her mum holds hope that she will ‘get back to her normal’. Anna's vision of her son's future was less clear:

He needs support in staying in touch with reality in the world and … where do you find a job that does that, where you know, that you can really contribute and do something that uses your mind and … and the things that you’re good at, but just gives you, helps you with that grounding and a bit of support. I don’t … I don’t know that that exists.

But life went on, and the parents were determined to support it.

##### Growth and me

Most felt they had grown from their experiences. Fatima and Emma talked of their preconceptions about mental health having been challenged:

Nobody could have pre‐warned me that this was going to happen in my home, because I would have sort of thought, it only happens to bad families. But it doesn't, it happens to good families as well. (Emma)

Anna used her been able to use her own previous lived experiences positively to support her son. Jackie had been taken out of her ‘comfort zone’ and became ‘stronger’. For Fatima, ‘After seeing what she [my daughter] went through, I completely changed myself. It's like completely different person’. For Jackie and Fatima, a deep maternal love and connection had been reinforced, and all but one felt their family was stronger.

All had been profoundly affected and the interview had been the first opportunity to talk. Challenges tumbled out through vivid retelling; Fatima described her daughter's piercing screams, while Emma and Jackie were stunned when their children reported that the voices said they had been raped. Peter's struggle was to both acknowledge his son's experiences as ‘real’ and ‘true’ and to help him move forward. The experience was summed up by Emma:

And even now I’m still not the person I was, even though they [my children] say, ‘mam, you’re a great mam and you do everything and you do too much’ and da da da. And yeah, it’s great to hear but it’s because I still want to be the person that I was. But don’t tell everybody it ages you about twenty years, But you’ve still got to have your sense of humour, never lose that.

## DISCUSSION

This study set out to explore how young people and parents had made sense of voice‐hearing in the family context. It highlighted young people's struggles to reconcile their experiences within themselves, their wish for control, ‘normality’ and to move forward. Parents saw the voices as separate to their child, tried to protect them, and came to an acceptance and hope for the future amidst continued uncertainty. Shame, isolation, hope, and pragmatism ran through parental and young people's accounts, though tensions, such as autonomy versus involvement, were also apparent.

Rather than the high levels of conviction of Kapur et al. (2014), results with the young people here show sense‐making as a dynamic and shifting work in progress. The young people had explored ideas around their voices but had found them mostly lacking. They reached for understandings for their experiences as a whole—not just voices—particularly those seen as more socially acceptable or ‘normal’, such as ‘stress’ or anxiety. Similar to adult studies, the relational themes of agency and control were central, though personifying (Holt & Tickle, 2014) or listening to the voices (Longden, 2017) less so. In contrast to Parry and Varese (2020), power increased, and distress decreased, with pragmatic acceptance and ‘getting on with life’ (Sarah), rather than understanding. While all the young people were engaged in a process of sense‐making, arriving at a destination was not as important as the adult literature suggests (British Psychological Society, 2017).

In parents’ accounts, voices presented as more actively present, as a malignant ‘other’ and an enemy to fight. Few had the explanatory clarity of the parents in Kapur et al. (2014), though they shared their struggles to understand and a heavy emotional load. In contrast to Parry and Varese (2021), biological explanations were prevalent, maybe as more visible, easier to comprehend, or culturally ‘acceptable’ than alternatives.

Families were not seen by the young people here as pivotal to their sense‐making. Where family was valued, it was as a safe and non‐enquiring space where they could be ‘normal’ and *did not* have to talk about voices. This chimes with Morrison et al. (2020) who highlighted that young people in first episode psychosis (FEP) services can be reluctant for family to be involved. However, in line with Parry et al. (2021), family stories and influences had clearly been absorbed, even if they were not explicitly acknowledged. Supporters that were most valued were often parents that they did not live with, siblings, extended family and friends. Health professionals were a containing and private space for exploration that respected young people's autonomy, echoing Byrne et al. (2020) on the value young people experiencing FEP place on having someone to talk to.

Shame, secrets, and isolation are a common feature of voice‐hearing accounts (Woods, 2017) and these are no exception. Here they tangle together with cultural and systemic influences such as fear of judgement and stigma (Parry et al., 2021). The strongest common influence between parents and young people, though contested in different ways, was ‘voices = bad/mad’. In line with developmental perspectives on adolescent individuation from the family (e.g. Anderson & Fleming, 1986), young people wanted to assert their independence and parents had to flex around this. Parents talked, listened, soothed, laughed, were there 24/7, and then got out of the way again when needed. They were pragmatic and cautiously protective while supporting their child's wider goals and plans, quietly celebrating everyday wins, and carrying hope.

This is one of only a small number of studies to look at how young people and parents make sense of voice‐hearing in the family context. Being a clinical sample from a range of settings, and not being diagnosis based, is a strength of the research. Limitations include that the sample was predominantly female and White British, which does not reflect clinical populations; the National Clinical Audit of Psychosis (Royal College of Psychiatrists, 2021) reports from case note sample that 62% of those in EIP services are male and 64% White British.

### Clinical and research implications

This study supports the case for further research on interventions that adapt therapy for young people, such as in reflecting their developing sense of identity and their social context (Jolley et al., 2018). It suggests therapeutic approaches that respect autonomy, strengthen control, and enable young people to hold uncertainty while getting on with life are important. The findings around isolation and connection reinforce the importance (and absence) of connecting with others with similar experiences. This is a core feature of the Hearing Voices approach (Corstens et al., 2014) that is lacking in many mainstream services.

Parents’ isolation is palpable and there is a clear need to help them process their own experiences and to develop their understanding of their child's experiences, including how best to support them. Perhaps, with better support, young people would feel they could draw more on family. None of the parents, and only one young person, talked of having had family intervention, which some studies in FEP (Byrne et al., 2020) report as being a positive, if difficult, experience for those who did take it up.

Research in a range of cultural and family contexts is needed to reflect the diversity of individuals’ experiences and beliefs and to tailor approaches accordingly. This study benefited from young people and parents being interviewed separately to elicit their individual experiences. Further research within family units could elucidate the dynamic nature of sense‐making.

## CONCLUSION

This research sets out to explore how young people and parents had made sense of voice‐hearing in the context of the family. Few had made sense of their experiences in any clear form, but this was not a prerequisite to feeling in control and pragmatically getting on with life. Young people valued family as a safe, non‐enquiring space to be ‘normal’ and *not* to talk. While all had been, and continued to be, challenged by their experiences, an energy, strength, and hope ran through their accounts.

## CONFLICT OF INTEREST

All authors declare no conflict of interest.

## AUTHOR CONTRIBUTION

**Claire Mayer:** Conceptualization (equal); Data curation (equal); Formal analysis (equal); Investigation (equal); Methodology (equal); Project administration (equal); Writing—original draft (equal). **Guy Dodgson:** Conceptualization (equal); Supervision (equal); Writing—review & editing (equal). **Angela Woods:** Conceptualization (equal); Methodology (equal); Resources (equal); Supervision (equal); Writing—review & editing (equal). **Ben Alderson‐Day:** Conceptualization (equal); Methodology (equal); Resources (equal); Supervision (equal); Validation (equal); Writing—review & editing (equal).

## Supporting information

Supinfo S1

## Data Availability

Data are not available due to privacy/ethical restrictions. Participants were not asked to consent to sharing of their interview data beyond the research team.
