# Supplementary material for: “Figuring out how to be normal”: Exploring how young people and parents make sense of voice‐hearing in the family context
Source: Psychol Psychother. 2022 Jan 20;95(2):600–14. doi: 10.1111/papt.12381 (PMC9303802; doi:10.1111/papt.12381)

**Supplementary materials**

1. **Interview schedule - young people**

| **PREAMBLE**  “I need to check before we start that you are happy to talk with me today.”  *[Establish whether young person verbally consents / assents]*  “I also need to remind you that what you say is confidential to us, unless I feel that something you say means you or others are at risk.”  “Do you have any questions for me at this stage?”  “I’m going to be asking some questions about the different kinds of experiences you’ve had with voices that other people cannot hear, and how you and your family have approached this.”  “Some people find the term “hearing voices” a useful way to describe their experience but you may have other words they use. Do you have words you would like me to use or is ‘hearing voices’ OK?”  “People sometimes worry about talking about this. Do you have any concerns? Is it OK to ask some questions about it?”  *[Establish participants are happy to talk about this]*  “There aren’t any right or wrong answers to what I’m going to ask. We’re going to be talking for around 60 minutes. If you would like to stop at any point, take a break, not answer any of the questions or would prefer to talk about something else, then it is absolutely fine to do that.”  *[Agree stopping rule or signal]* |
| --- |
| 1. **Voice hearing and sense making**   “Could you tell me a bit about the voices your hear”  Follow ups:   - *First time you remember having voice hearing experiences?* - *How were things going at the time?* - *What was life like for you?* - *How were you feeling?* - *Can you remember who you first told about it?* |
| 1. **Sense making**   “Do you have any ideas about who or what the voices you hear are? Do you have any ideas why you hear them?”  Follow ups:   - *Have they been caused by something that has happened to you, do you think?* - *What do the voices mean to you?* - *How do you feel about the voices?* - *What do you agree / disagree on?* - *Who has been important in helping you think about why you hear voices?* |
| 1. **Telling family**   “We’re going to think about how you told your family about your voice hearing experiences. Can you tell me who the important people in your family are?” [using family paper sculpt here]  “Do you remember the first time you remember talking to one of you family about it”  Follow ups:   - *Who was it?* - *How did you come to tell them?* - *How come you told them?* - *How did you feel before / after?* - *Was it helpful / unhelpful for you?* - *What happened next?*   “What about the other members of your family?” [go through family paper sculpt to talk about and move family members]  Follow ups:   - *What was that like?* - *How come you told them?* - *How did you feel before / after?* - *Was it helpful / unhelpful for you?* - *What happened next?*   *“*Do you and your family agree / disagree on why you hear voices?” |
| 1. **Impact on relationship with family**   “I’d like you to each have a look at these [blob pictures] and chose which of them you think best describes the relationship with your family when it comes to the voices. Tell me about the picture you’ve chosen”  Follow ups:   - *How / has voice hearing affected your relationship with family members?* - *Is it better / worse / different?* - *Has if affected how you feel about each other?* - *What has been positive about it?* - *What might have been more negative about it?* |
| - **Talking about it together as a family**   “What’s it like talking to your family about it now?” [using family sculpt as prompt]  Follow ups:   - *Has this been easy / difficult?* - *What makes it easier / more difficult?* - *Has it changed over time?* - *Are there things that you find more difficult to talk about together? How come?* - *Has anything helped with talking together?* - *Are there people in your family you don’t talk to about it?* - *Has talking about it changed how you feel about it?* - *Has your family had an impact on why you think you hear voices?* |
| 1. **Advice**   “Finally, what advice would you have for other young people and their families who are trying to understand voice hearing?” |
| ***DEBRIEF***  “Thank you for sharing your experiences with me; it’s been a privilege to hear about them”.  Take through debriefing sheet:   - Next steps: we listen to all the interviews and pull out the main things from each - Interim results will be shared with them if would like, to comment on - They may also be invited to an event with other young people and parents to discuss the findings - Final results will also be shared with them if they wish - Where can get further support   “Do you have any questions you would like to ask me? It could be about anything we’ve discussed or what happens next with what we’ve talked about”  Check in: How did you find talking about things? Is there anything you found particularly difficult? Is there anything you’d like to talk about now? “Do you feel OK to go home?”  “Thank you” |

1. **Interview schedule – parents**

| **PREAMBLE**  “I need to check before we start that you are happy to talk with me today.”  *[Establish whether they verbally consent / assent]*  “I also need to remind you that what you say is confidential to us, unless I feel that something you say means you or others are at risk.”  “Do you have any questions for me at this stage?”  “I’m going to be asking some questions about the different kinds of experiences your child has had with voices other people cannot hear, and how they, you and the family have approached this.”  “Some people find the term “hearing voices” a useful way to describe their experience but you may have other words they use. Do you have words you would like me to use or is ‘hearing voices’ OK?”  “People sometimes worry about talking about this. Do you have any concerns? Is it OK to ask some questions about it?”  *[Establish participants are happy to talk about this]*  “There aren’t any right or wrong answers to what I’m going to ask. We’re going to be talking for around 60 minutes. If you would like to stop at any point, take a break, not answer any of the questions or would prefer to talk about something else, then it is absolutely fine to do that.”  *[Agree stopping rule or signal]* |
| --- |
| 1. **Voice hearing experiences**   “Could you tell me a bit about the voices your child hears”  Follow ups:   - *First time you remember knowing they had voice hearing experiences?* - *How were things going at the time?* - *What was life like for them?* - *How were they feeling?* - *Can you remember who you first told about it?* |
| 1. **Sense making**   “Do you have any ideas about who or what the voices your child hears are? Do you have any ideas why they hear them?”  Follow ups:   - *Have they been caused by something that has happened to them, do you think?* - *What do the voices mean to you?* - *Do you see any connections between your own and your child’s experiences?* - *How do you feel about the voices?* - *Who has been important in helping you think about why your child hears voices?* |
| 1. **Telling family**   “We’re going to think about your child’s family and how they’ve been about your child’s voice hearing experiences. Can you tell me who the important people in the family are?” [using family paper sculpt here]  “Do you remember the first time you remember your child talking to you about it”  Follow ups:   - *How did they come to tell you?* - *How did you feel?* - *How did you react?* - *What did you make of it?* - *How did you / they feel before / after?* - *Do you think it was helpful / unhelpful for you / them?* - *What happened next?* - *What’s it like now?*   “What about the other members of your family? How have they been?” [go through family paper sculpt to talk about and move family members]  Follow ups:   - *How come they know?* - *How do they feel?* - *How have they reacted?* - *Has that been helpful / unhelpful?*   “Do you and your child agree / disagree on why your child hears voices? What about other members of the family?” |
| 1. **Impact on relationship with family**   “I’d like you to each have a look at these [blob pictures] and chose which of them you think best describes the relationship with your child when it comes to the voices. Tell me about the picture you’ve chosen”  Follow ups:   - *How / has voice hearing affected your relationship with your child? What about your child’s relationship with family members? Your relationship with others?* - *Is it better / worse / different?* - *Has if affected how you feel about each other?* - *What has been positive about it?* - *What might have been more negative about it?* |
| 1. **Talking about it together as a family**   “What’s it like talking as a family about it now?” [using family sculpt as prompt]  Follow ups:   - *Who talks about it / doesn’t talk about?* - *Has this been easy / difficult? What makes it easier / more difficult?* - *Has it changed over time?* - *Are there people in your family you don’t talk to about it?* - *Has talking about it as a family changed how you feel about it?* - *Has the family had an impact on why you think your child hears voices?* |
| 1. **Advice**   “Finally, what advice would you have for other young people and their families who are trying to understand voice hearing?” |
| ***DEBRIEF***  “Thank you for sharing your experiences with me; it’s been a privilege to hear about them”.  Take through debriefing sheet:   - Next steps: we listen to all the interviews and pull out the main things from each - Interim results will be shared with them if would like, to comment on - May also be invited to an event with other young people and parents to discuss the findings - Final results will also be shared with them if they wish - Where can get further support   “Do you have any questions you would like to ask me? It could be about anything we’ve discussed or what happens next with what we’ve talked about”  Check in: “How did you find talking about things? Is there anything you found particularly difficult? Is there anything you’d like to talk about now? Do you feel OK to go home?”  “Thank you” |

1. **Sample family sculpts**


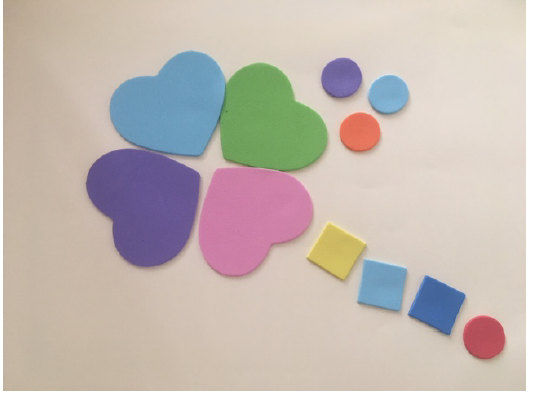


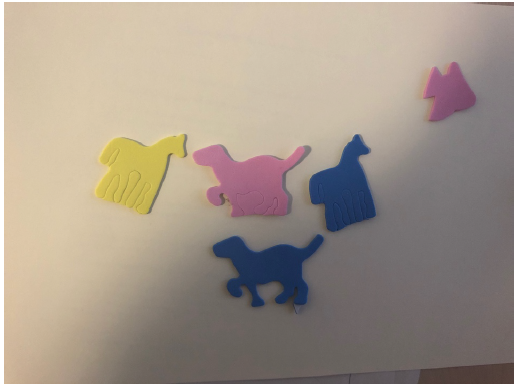

Supplement: Supplementary file 1 — Supinfo S1 [file PAPT-95-600-s001.docx]
